# Examining the Relationship Between the Use of a Mobile Peer-Support App and Self-Injury Outcomes: Longitudinal Mixed Methods Study

**DOI:** 10.2196/21854

**Published:** 2021-01-28

**Authors:** Kaylee Payne Kruzan, Janis Whitlock, Natalya N Bazarova

**Affiliations:** 1 Center for Behavioral Intervention Technologies Northwestern University Chicago, IL United States; 2 Bronfenbrenner Center for Translational Research Cornell University Ithaca, NY United States; 3 Department of Communication Cornell University Ithaca, NY United States

**Keywords:** self-injury, mobile apps, peer support, mHealth

## Abstract

**Background:**

Many individuals who self-injure seek support and information through online communities and mobile peer-support apps. Although researchers have identified risks and benefits of participation, empirical work linking participation in these web-based spaces to self-injury behaviors and thoughts is limited.

**Objective:**

This study aims to investigate the relationship between behavioral and linguistic traces on a mobile peer support app and self-injury outcomes.

**Methods:**

Natural use data and web-based surveys (N=697) assessing self-injury outcomes were collected from 268 users (aged 13-38 years; median 19; 149/268, 55.6% female) of a mobile peer-support app for 4 months. Participants were identified as having posted self-injury content using an internal classifier. Natural log data was used to predict self-injury outcomes in a series of multilevel logistic and linear regressions.

**Results:**

Greater engagement on a mobile peer-support app was associated with a decreased likelihood of self-injury thoughts (odds ratio [OR] 0.25, 95% CI 0.09-0.73) and fewer intentions to self-injure (*b*=−0.37, SE 0.09), whereas posting triggering content was associated with an increased likelihood of engaging in behaviors (OR 5.37, 95% CI 1.25-23.05) and having self-injury thoughts (OR 17.87, 95% CI 1.64-194.15). Moreover, viewing triggering content was related to both a greater ability to resist (*b*=1.39, SE 0.66) and a greater intention to self-injure (*b*=1.50, SE 0.06).

**Conclusions:**

To our knowledge, this is the first study to connect naturally occurring log data to survey data assessing self-injury outcomes over time. This work provides empirical support for the relationship between participation in online forums and self-injury outcomes, and it articulates mechanisms contributing to this relationship.

## Introduction

### Background

Self-injury—the deliberate, self-inflicted damage of body tissue [1]—is a common and concerning behavior estimated to affect between 17% and 37% of adolescents [2,3]. Part of a larger spectrum of self-harming behavior, nonsuicidal self-injury (NSSI) does not include suicidal intent but is often comorbid with other mental health challenges, besides being a leading risk factor for future suicidal thoughts and behaviors [4,5]. For a variety of reasons, including stigma and a lack of readiness to change, many individuals who self-injure do not disclose their behavior to anyone, impeding potential for intervention. Despite hesitancy to disclose self-injury offline, individuals discuss their experiences with self-injury relatively openly on the web [6-9]. Prior work has identified a number of benefits and risks of using online forums to seek self-injury-related support or information [7-10]. Implicit in this line of research is the assumption that web-based venues can meaningfully impact self-injury thoughts and behaviors. However, to date, only a few empirical studies have examined the effects of participation in online communities on self-injury outcomes [11,12], and the bulk of this literature has been descriptive, cross-sectional, and focused on relatively small samples.

Mobile apps are an increasingly common way for individuals to access self-injury communities and resources, and these technologies can be used to deliver interventions [13-16]. Several apps show promise in reducing self-injury behaviors [14,15]. However, by and large, the efficacy of most mobile apps for mental health is untested [17]. Advances in computational techniques enable researchers to track patterns of behavior on the web to predict mental health status and future risk [18,19], but such methods have not yet been robustly applied to understand contexts that contribute to and predict self-injury behaviors.

This work employs a mixed methods approach to address the gap in knowledge on the relationship between web-based self-injury support activity and self-injury outcomes. Specifically, we combine computational and survey methods to investigate the relationship between language and behaviors on a mobile peer support app, on the one hand, and self-injury behaviors, thoughts, intentions to self-injure, and ability to resist self-injury urges over time, on the other hand. This work provides empirical support for the relationship between participation in online forums and self-injury outcomes and articulates mechanisms contributing to this relationship.

### Functions of Self-Injury

Self-injurious behaviors can serve a variety of intrapersonal and interpersonal functions [20,21]. Self-injury is most often enacted to regulate emotions, and this function is evidenced in affect modulation following self-injury episodes through laboratory and ecological momentary assessment (EMA) studies [20]. In contrast, interpersonal functions include signaling relational distress, soliciting social support, or escaping undesired interpersonal situations [22].

A functional understanding of self-injury is useful when interrogating the relationship between web-based activity and self-injury because it provides guidance on factors temporally associated with the behavior. Interpersonal functions are of particular interest because social and relational factors are likely salient in web-based spaces where people provide and receive peer support [23] and may play a role in the initiation, maintenance, and cessation of self-injury for adolescents. Relational factors (eg, the volume of support exchanged, group affiliation) also merit further attention as they could provide insights into participatory risks (eg, normalization) [10,12,24,25].

### Potential Risks and Benefits of Web-Based Activity

The potential benefits of online peer-to-peer support networks for individuals with mental health conditions include receipt of social support, validation, an increased sense of belonging [6,26], and the ability to narrate, and reflect on personal experiences (eg, share experiences for personal clarity) [6]. Web-based venues may also provide a useful distraction to assuage self-injury urges [27] and facilitate the receipt of *just in time* support [28].

Other studies substantiate a growing concern over the potential for adverse effects. Risks of exchanging online support for self-injury include reinforcement of the behavior, excessive focus on emotional suffering and rumination, and exposure to triggering content [10,28,29]. Research has shown that participation in online communities can result in the normalization of self-injury and an overreliance on the community for support [30]. Individuals may also feel the need to maintain an *injury identity* [31] where the behavior is seen as critical for community membership and enacted to validate the severity of individuals’ experiences [27,32,33].

There is also concern that exchanges in online communities may downplay the serious consequences of the behavior [28] and discourage professional help, either explicitly or implicitly, through sharing past negative experiences [30,34]. A risk that has received much attention recently is exposure to graphic and triggering content. Some members of online self-injury communities report that graphic content curbs the urge to injure because it presents them with severe cases and can dissuade them from future self-injury acts [9,33]. However, others describe seeking out content in online forums to trigger self-injury urges [7,25,35].

Regarding how participation in online communities modulates self-injury behavior, the evidence is mixed. Murray and Fox [35] found that just over 40% of 79 respondents reported that participation in a web-based discussion group reduced their self-injurious behavior, whereas 11% reported that it initiated behavior. Harris and Roberts [7] found a similar split in evidence. Other studies have shown that greater self-injury content exposure is associated with greater self-injury engagement [36]. Internet addiction [37,38] and cyberbullying [39] have also been associated with self-injurious behaviors. The largely cross-sectional nature of the relationships studied makes discerning temporal sequencing difficult.

### Characteristics and Contexts Likely to Influence Self-Injury Outcomes

When thinking about characteristics and contexts associated with self-injury outcomes, 2 additional lines of work can be informative: (1) diary and EMA studies and (2) computational mental health research.

#### Diary and EMA Studies

A recent review of self-injury EMA studies identified emotional, cognitive, and social contexts associated with NSSI, motives that lead to NSSI, and mechanisms that influence or predict NSSI [40]. Several studies have found that self-injurious behaviors are flanked by changes in affect, which can be apparent up to 15 hours before NSSI acts [41,42]. In general, negative affect often precedes self-injury [42-44], whereas increases in positive affect and decreases in negative affect follow [44,45]. Furthermore, NSSI thoughts have been linked to sadness and anxiety [46,47], whereas NSSI behaviors have been associated with rejection and anger [46,48]. Affective instability (sometimes referred to as affective lability)—the tendency to experience emotions in a dynamic manner with extreme shifts in emotion lasting up to a few days—adds another emotion-linked dimension to self-injury risk, with research showing that compared with individuals with no self-injury history, individuals who self-injure experience more affective instability [49,50]. Frequent shifts in emotional intensity and valence have been associated with more NSSI episodes [49].

Cognitive states and patterns are also empirically associated with self-injury [51]. For example, rumination and fluctuations in the intensity of ruminative thinking (known as rumination instability) are theorized to play a key role in NSSI [52,53]. Selby et al [54] found that rumination instability and fluctuations in negative affect, especially sadness, interacted to predict daily reports of NSSI in a 2-week EMA study. Similarly, Hughes et al [43] found that repetitive negative thinking and negative affect predicted NSSI thoughts and behaviors and amplified the effects of anxiety and overwhelm.

Finally, contextual factors, such as interpersonal conflict and feelings of rejection, are powerful predictors of same-day self-injury thoughts and behaviors. Interestingly, a study found that although the act of revealing NSSI to others is associated with greater perceived social support, perceiving support increases the likelihood of self-injury on the following day [55]. Therefore, interpersonal reinforcement is a risk factor for self-injury behaviors.

Although EMA studies provide insights into the complex temporal interplay between context, cognition, emotion, and behavior, by accessing data *in real time*, these methods often rely on self-reports, limited by participant awareness, and may not be apparent, or readily accessible, for individuals struggling with emotion regulation [41]. Methods that leverage modern computational and algorithmic capabilities can help produce a robust understanding of the complex sequencing and interplay between emotion, cognition, and behavior by analyzing log data in online communities.

#### Computational Mental Health Research

Previous studies have shown that behavioral patterns and linguistic features of posts in web-based communities can distinguish between people with and without a number of conditions [56-58] and can be used to infer risk [59,60]. Much of these studies have combined behavioral measures such as posting frequency, with linguistic features such as language use or themes embedded in published content. In several studies, individuals with depression showed greater negative emotion, higher self-attentional focus, and increased relational and medicinal concerns in posts than those without known depression [56]. Behaviorally, depressed members showed less engagement, social activity, and reduced reciprocity [57].

Suicidal ideation has also been associated with self-attentional focus and reduced social engagement and expressions of hopelessness, anxiety, impulsiveness, and loneliness [18]. Temporal patterns can also be discerned using this methodology. For example, research has found an increase in posting activity before a suicidal attempt, along with increases in anger and sadness in the posts. Conversely, declines in activity, anger, and sadness follow suicide attempts, at which point attempters’ levels of activity and emotions mirror those of nonattempting peers [59]. In summary, the computational techniques employed in these studies have provided impressive predictive accuracy [61] and may be useful in disentangling the complex interplay of thought, emotion, and behavior that leads to self-injury.

### Objectives

This study aims to leverage the strengths of the methodologies mentioned above—rich, naturally occurring web-based data and self-report survey data over 12 weeks—to explore how self-injury behaviors and thoughts are related to activity and language use of self-injurious users on the TalkLife platform, a free mobile app designed for young people with a variety of mental health concerns. The platform uses a crowdsourced peer-support model to provide users with affordable and timely support. Self-injury outcomes are modeled (eg, behavior, thoughts, intentions, ability to resist) as a function of web engagement and language manifested in content. We explore 2 dominant questions: What behavioral (research question [RQ] 1) and language (RQ2) patterns are associated with self-injury behaviors, self-injury thoughts, intentions to self-injure, and ability to resist self-injury? These 2 questions are further broken down into the following subquestions:

- *RQ1a*: What is the relationship between *activity level* on TalkLife in the preceding week and self-injury outcomes (behavior, thoughts, intentions, ability to resist, and frequency) in the subsequent reporting period?
- *RQ1b*:What is the relationship between *viewing triggering content* on TalkLife in the preceding week and self-injury outcomes (behavior, thoughts, intentions, ability to resist, and frequency)?
- *RQ1c*: What is the relationship between *posting triggering content* on TalkLife in the preceding week and self-injury outcomes (behavior, thoughts, intentions, ability to resist, and frequency)?

Next, we examine language that may be predictive of self-injury outcomes. On the basis of interpersonal models of self-injury [62,63], we probe the association between community involvement and themes of family and friends and self-injury outcomes. As a proxy for identification with the community, we assess affiliative language as in previous work [64].

- *RQ2a*: What is the relationship between using an *affiliative language* in the preceding week and self-injury outcomes (behavior, thoughts, intentions, ability to resist, and frequency)?
- *RQ2b*: What is the relationship between *mentions of family and friends* in the preceding week and self-injury outcomes (behavior, thoughts, intentions, ability to resist, and frequency)?
- *RQ2c*:What is the relationship between *specific emotional states* (eg, positive and negative emotions, rumination) in the preceding week and self-injury outcomes (behavior, thoughts, intentions, ability to resist, and frequency)?

## Methods

To understand how the mobile app’s use is related to self-injury outcomes, we employed a mixed-methods approach utilizing surveys and naturally occurring log data over 4 months; 3 types of data are included in the analyses: (1) responses to surveys, (2) behaviors on the platform, and (3) language use in posts and comments. A description of data acquisition, relevant measures, and treatment of these measures follows.

### Survey Data

Surveys were issued on a rolling basis for 12 weeks. The first and last surveys were administered on October 25, 2018, and January 17, 2019. Survey administration was triggered internally by a classifier identifying suspected self-injury content. Once participants’ posts were flagged, they received a prompt to answer surveys once a week for the duration of the study period. Due to this method, the total duration of the study for any given participant varied. Participants could opt out of weekly surveys at any point.

The final data set was constrained to participants who had completed at least two surveys and corresponding behavioral and language data were extracted based on this criterion. Participants who did not complete basic demographics or who did not complete at least one self-injury outcome variable in a survey were removed from the sample. The number of surveys participants completed varied (mean 2, SD 1.20; range 1-10 surveys), as did the time between surveys (mean 1.74, SD 2.15 weeks; range 1-11.6 weeks). The total number of participants included in the final analyses was 268 with 697 survey observations. Our institutional review board approved all study procedures and data security measures.

Surveys included 9 items that were administered weekly with a question to address the (1) presence of self-injury behaviors, (2) frequency of self-injury behaviors, (3) presence of self-injury thoughts, (4) intensity of self-injury intentions, and (5) ability to resist. Additionally, there were items for past experience with therapy, age of first self-injury, and demographics, including age (how old are you?), race (what is your race?), and gender (what is your gender?).

Self-injury items (1-5 above) were treated as dependent variables and demographics (age, race, and gender) were included as covariates in all models. Response categories for self-injury behavior and thoughts were binary: *Yes* or *No*. The ability to resist urges and intentions to injure were both answered on a 5-point scale ranging from 0 (not at all strong) to 4 (very strong) and treated as continuous variables. The frequency of self-injury behaviors required participants to enter a number; 4 participants reported engaging in self-injury at highly improbable rates (>500 times). To correct for these outliers, we reduced these values to 100. Thus, the final self-injury frequency variable ranged from 0 to 100.

### Behavioral Data

Deidentified behavioral data for participants meeting the above criterion (2 or more surveys) were sourced with license and consent from the TalkLife platform. This included metadata and original posts and comments. Given that weekly surveys referred to self-injury activity in the previous week, behavioral data at 1 week before each weekly survey were extracted as the primary data for prediction. In addition to controlling for demographics, we controlled for differences in time (relative to survey number) in all analyses because of the survey administration’s rolling basis.

We focus on several measures in analysis: (1) activity level (operationalized as averages of posts, gifts, reactions, comments, likes, and users followed in the previous week), (2) posting triggering content (operationalized as the number of posts a user published with trigger warnings), and (3) viewing triggering content (operationalized as the number of times a user dismissed trigger warnings when looking at others’ posts). All of these variables were averaged at the day level and log-transformed to restore normality because of their high positive skew.

Given that variance in behavior has proven to be a meaningful independent predictor of mental health in previous work [56], 2 measures that capture fluctuations in activity were also included: variance and rate of change. The variance was computed at the day level for all behavioral measures in the week before a given survey. This measure was computed as *σ*^2^, where the mean activity level in a given week (*μ*) was subtracted from the activity on a given day (*χ*) for all days of the week, this was squared, summed (Σ), and divided by 7 (N).


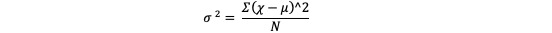


This variance measure provides a sense of how an individual’s log data (eg, activity, publishing, and viewing triggering content) is distributed over the week. A high variance score is a proxy for instability or more change in activity over the course of the week (eg, users are very active one day, have no activity the next, and then are moderately active). Next, we computed a *change* score to capture the magnitude of change between proximal behavior (behavior in the week before the survey) and more distal behavior (the remaining time between surveys). This measure was adapted from previous work and is sometimes called the *rate of change* [56]. To account for differences in the amount of time in the remaining period, we averaged to the day level, that is, change (Δ) was equal to the mean activity in a week before a survey (A)—the mean activity in the remaining period (B). A positive change score indicates that there was more activity on a daily basis in the week before the survey, whereas a negative difference indicates more activity in the distal period.

Δ = *A* – *B*

This change variable was entered into the models as continuous, including negative and positive values. Thus, the interpretation of this variable should be as a *rate of change* or the magnitude of difference between the 2 periods. A large value indicates a large difference between activity in the week before a survey, relative to the time before, whereas a small value signals relatively similar activity in these 2 periods.

The final variables for the behavioral data include: (1) activity, (2) trigger posts, (3) trigger dismiss, (5) variance (×3), and (6) change between proximal and distal activity (3).

### Language Data

All posts and comments made by participants within the study period were preprocessed and run through the Linguistic Inquiry and Word Count (LIWC) program, a psycholinguistic text analysis tool that is frequently employed in research on mental health [65].

Relevant dimensions were identified from the initial literature review, including: (1) affect (eg, positive emotion and specific negative emotions [sadness and anger]), (2) social or relational (eg, mentions of family or friends), (3) affiliative language (eg, affiliation and *we* pronouns based on [64,66]), (4) self-focus (eg, *I* language), (4) rumination (a composite score of negative emotion and focus on the past), and (5) efficacy (a composite score of focus present, future, and certain language as in a study by Bliuc et al [64]).

As with the behavioral data, variability and change were computed for select language dimensions based on previous research. On the basis of the emotional cascade model [54] and empirical findings on the role of instability of rumination and negative affect before self-injury episodes [52,53], we include variance and change for rumination, positive emotion, sadness, and anger.

### Sample Characteristics

Descriptive statistics for the survey data are provided in Table 1. The sample consisted of 268 participants who were mostly female (149/268, 55.6%), White (164/268, 61.2%), and were around the age of 19 years (median 19; range 13-38). Over 40% of participants reported having received therapy at some point during the study period (113/268, 42.2%), and the median age at first self-injury was 14 years (range 5-37). On average, participants were registered on TalkLife for about 10 months (SD 12.8) and had posted a median number of 49 posts (mean 147.66, SD 309.72) and 360 comments (mean 1775.28, SD 4092.56).

**Table 1:** Participant characteristics (n=268).

Characteristic		Value, n (%)
**Gender**		
	Male	83 (30.9)
	Female	149 (55.6)
	Transgender or nonbinary^a^	36 (13.4)
**Race**		
	White	164 (61.2)
	Black	13 (4.9)
	Asian	43 (16.0)
	Other^b^	47 (17.5)

^a^The response options of transgender male-to-female (n=2), transgender female-to-male (n=8), do not identify as male or female (n=12), and not sure (n=14) were combined because of small cell sizes.

^b^The response options of American Indian or Alaskan Native (n=6), Native Hawaiian or other Pacific Islander (n=3), and other (n=38) were combined because of small cell sizes.

During the 4 months of this study, 48.5% (130/268) of participants reported self-injury behaviors, and 84.7% (227/268) reported having self-injury thoughts. Of those who reported injuring, the median weekly frequency was 3 times. Overall, 79.5% (213/268) of participants reported having thoughts of self-injury without engaging in self-injury behavior, whereas only 2.6% (7/268) of participants reported self-injury behaviors without also reporting self-injury thoughts.

### Data Analysis Plan

Before analysis, diagnostic tests were run to determine appropriate modeling and the need for further data transformation. As mentioned above, highly skewed predictor variables (activity, trigger posts, and trigger views) were corrected through log-transformation. Self-injury frequency was the only response variable to be abnormally distributed and was thus also log transformed. Multicollinearity was assessed for all variables in relation to each dependent variable, using the R package mctest. The highest variance inflation factor (VIF) factor was consistently reported for rumination (8.92-9.58), followed by self-referent language (4.22-5.18). The mean VIF for each outcome was acceptable (self-injury behavior, 3.48; self-injury thoughts, 3.43; intentions to injure, 3.32; ability to resist, 3.33; and self-injury frequency, 3.37). As multicollinearity was not detected, we proceeded with analyses without excluding any variables at the outset.

The relationship between TalkLife activity and self-injury outcomes was analyzed using multilevel analysis to account for the data’s nested structure. Survey responses, log data, and language data were nested at the participant’s level; therefore, we included random effect of participant in all analyses. A total of 5 models were run to predict (1) self-injury behavior, (2) self-injury thoughts, (3) ability to resist the urge to injure, (4) intentions to injure, and (5) behavioral frequency. Logistic regressions predicting behavior and thoughts were analyzed using the R lme4 package, and linear models predicting the ability to resist, intentions, and frequency were analyzed with the nlme package. All models were adjusted for demographics (age, gender, and race) and time points.

Given this work’s exploratory nature, we began with full models including the 31 variables described above (4 control variables, 3 log variables, 10 language variables, 8 variance measures, and 8 change scores). These full models were subsequently reduced via backward variable selection. The logged coefficients were exponentiated for easier interpretation for the binary dependent variables (behaviors and thoughts). We report the significant results in the following section.

## Results

### Self-Injury Behavior

In terms of behaviors on the web, the odds of engaging in self-injury behavior increased with the number of triggering posts published in the week before the survey. For every additional unit increase in the log of triggering posts, the odds of engaging in self-injury behavior increased nearly five-fold (OR 5.37, 95% CI 1.25-23.05; *P=*.02). In addition, as the rate of change for viewing triggering content increased (ie, viewing more triggering content in the week before the survey compared with the distal period), the odds of self-reported self-injury behavior decreased (OR 0.81, 95% CI 0.68-0.98; *P=*.03). In other words, for every 1-unit increase in change between the number of times individuals viewed triggering content in the week of a survey, the likelihood of self-injury behavior decreased by roughly 20% relative to the period before. No significant relationships were found between self-injury behavior and language dimensions (Table 2).

**Table 2:** Self-injury behavior.

Self-injury behavior		B	SE	OR^a^ (95% CI)
Intercept		−0.07	0.64	0.93 (0.27-3.29)
**Behaviors on the web**				
	Trigger posts	1.68^b^	0.74	5.37 (1.25-23.05)
**Change**				
	Trigger views	−0.20^b^	0.10	0.81 (0.68-0.98)

^a^OR: odds ratio.

^b^The model was adjusted for demographics (age, gender, and race) and time point. Age and race were significant at *P=.*01.

### Self-Injury Thoughts

For behaviors on the web, activity level emerged as a significant predictor of self-injury thoughts. Greater active use of the platform (as indicated by posts, comments, and likes) was associated with lower odds of reporting self-injury thoughts (OR 0.64, 95% CI 0.45-0.90; *P=*.01). For every 1-unit increase in the log of activity, the odds of self-injury decreased by 36%. In contrast, the number of trigger posts published was positively related to self-injury thoughts—for every additional log unit increase in triggering posts, the odds of self-injury thoughts increased by a factor of 17.87 (95% CI 1.64-194.15; *P=*.02). The rate of change of posting triggering content also predicted self-injury thoughts; as this change decreased (ie, less activity in the week before the survey relative to the distal period), the odds of having self-injury thoughts increased (OR 0.29, 95% CI 0.10-0.86; *P=*.02). In terms of language, greater variation in ruminative language was associated with greater odds of self-injury thoughts (OR 1.15, 95% CI 1.02-1.29; *P=*.02); see Table 3 for additional details.

**Table 3:** Self-injury thoughts.

Self-injury thoughts		B	SE	OR (95% CI)
Intercept		1.78^a^	0.67	5.91 (1.59, 21.97)
**Web-based behaviors**				
	Activity	−0.45^a^	0.18	0.64 (0.45-0.90)
	Trigger posts	2.88^a^	1.22	17.87 (1.64-194.15)
**Change**				
	Trigger posts	−1.22^a^	0.55	0.29 (0.10-0.86)
**Variance**				
	Rumination	0.14^a^	0.06	1.15 (1.02-1.29)

^a^The model was adjusted for demographics (age, gender, and race) and time point. Race was significant at *P=.*01.

### Ability to Resist Self-Injury

For behaviors on the web, the number of trigger warnings dismissed was positively related to the ability to resist (*b=*1.39, SE 0.66; *P=*.03), that is, for every additional log unit increase in viewing trigger posts, the ability to resist self-injury also increased.

Several language dimensions also emerged as significant. In particular, the use of self-referent language (*I*) was negatively associated with the ability to resist (*b=*−0.07, SE 0.03; *P=*.01), whereas the use of *efficacy* language was positively associated with the ability to resist injuring (*b=*0.14, SE 0.06; *P=*.01). In addition, as the change score for positive emotional language increased (ie, more use of positive emotional language in the week before a survey relative to the distal period), the ability to resist also increased (*b=*.05, SE 0.02; *P=*.04). This means that the greater the magnitude of difference between positive language used in the week before a survey, relative to the period before, the more participants reported being able to resist urges. The variance in dismissing trigger warnings was negatively associated with the ability to resist such that lower variance (or more stability) across days in the week before a given survey was associated with greater ability to resist (*b=*−0.18, SE 0.08; *P=*.02). In contrast, greater variance in anger expression was associated with a greater ability to resist urges to self-injury (*b=*0*.*19, SE 0.09; *P=*.03); see Table 4 for additional details.

**Table 4:** Ability to resist self-injury.

Ability to resist self-injury		B	SE
Intercept		2.10^a^	0.24
**Web-based behaviors**			
	Trigger views	1.39^a^	0.66
**Language**			
	I^b^	−0.07^a^	0.03
	Efficacy	0.14a	0.06
**Change**			
	Positive emo	0.05^a^	0.02
**Variance**			
	Trigger dismiss	−0.18^a^	0.08
	Anger	0.19^a^	0.09

^a^The model was adjusted for demographics (age, gender, and race) and time point. Gender (*P=.*04) and race (*P=.*01) were significant.

^b^Self-referent language.

### Intentions to Self-Injure

We noted a negative association between activity and intention to injure (*b=*−0.37, SE 0.09; *P<*.001). In contrast, there was a positive association between the number of trigger warnings dismissed and intentions to injure (*b=*1.50, SE 0.06; *P=*.01). We also found that familial language was negatively associated with intentions to injure (*b=*−0.32, SE 0.22; *P=*.01). We noted a negative relationship between the change in dismissing trigger warnings and intentions to injure such that greater changes in viewing triggering content (ie, more viewing triggering content in the week before a survey compared with a distal period) were related to less intention to injure (*b=*−0.06, SE 0.03; *P=*.04); see Table 5 for additional details.

**Table 5:** Intentions to self-injure.

Intentions to self-injure		B	SE
Intercept		2.43^a^	0.26
**Web-based behaviors**			
	Activity	−0.37^a^	0.09
	Trigger views	1.50^a^	0.61
**Language**			
	Family	−0.63^a^	0.22
**Change**			
	Trigger views	−0.06^a^	0.03

^a^The model was adjusted for demographics (age, gender, and race) and time point. Age and race were significant (*P=.*02).

### Self-Injury Frequency

There was a positive relationship between the number of posts published with triggering content in the previous week and the frequency of self-injury behaviors reported in the following report period (*b=*0.45, SE 0.15; *P=*.002). A positive relationship also surfaced between *I* language and frequency of self-injury behaviors (*b=*0.02, SE 0.01; *P=*.04); see Table 6 for additional details.

**Table 6:** Self-injury frequency.

Self-injury frequency		B	SE
Intercept		0.59^a^	0.15
**Web-based behaviors**			
	Trigger posts	0.45^a^	0.15
**Language**			
	I^b^	0.02^a^	0.01

^a^The model was adjusted for demographics (age, gender, and race) and time point. Age was significantly different (*P=.*03).

^b^Self-referent language.

## Discussion

### Principal Findings

In this study, we employed survey responses and naturally occurring log data from a mobile peer-support platform to predict self-injury outcomes. This study fills an important gap in the research literature by connecting behavioral and language patterns to self-reported self-injury outcomes and offers new insights into the relationship between participation in online communities and self-injury.

### Key Findings on Web-Based Behavior

One of the primary aims of this work was to shed light on what specific behaviors may be beneficial or detrimental; much of the work on risks and benefits of participation in online communities has been qualitative and has not rigorously examined specific web-based activities [67,68]. This study’s findings are consistent with other studies suggesting that participation in online peer support forums may reduce self-injury thoughts by offering useful distraction and providing links to resources [9,27,33]. This study found that activity level predicted decreased thoughts and intent to injure. Participants who actively engaged in TalkLife—through posting content, liking, and generally interacting with others—were at lower odds of reporting self-injury thoughts and intentions. Activity did not, however, predict self-injury behavior or the ability to resist self-injury.

There are several possible explanations for these findings. The most direct explanation is that active use of TalkLife reduces self-injury thoughts and intent, which is in alignment with what the platform was designed to do. This is consistent with the work showing that individuals who engage in active use of social media derive important benefits, such as cultivating feelings of support, connections with others, and companionship [69]. Another possibility is that individuals active on TalkLife represent a self-selecting group with fewer than average day-to-day thoughts or urges to injure. These individuals may be in more advanced stages of recovery or may be qualitatively different from other users somehow. However, as previous work suggests that individuals who frequent online communities are often early in the stage-of-change process [70], it is more likely that users would report higher than average self-injury thoughts and behaviors.

Unlike being active on the app, posting triggering content was positively associated with self-reported self-injury thoughts and behaviors. In other words, although active use appears to be indicative of fewer thoughts and intentions to injure, the types of content posted—specifically content that has been labeled as triggering—predicted an increased risk of self-injury behaviors and thoughts. Given the nature of these analyses, it is not possible to infer the causal direction of this finding as triggering posts may have been published before or after self-injury thoughts or behavior. Nevertheless, the high temporal correlation of posting triggering content and reporting self-injury thoughts and behaviors suggests that it could be leveraged to check in on users and provide support at key junctures. The directionality of this relationship should be explored in future work.

Interestingly, viewing triggering content appears to be positively related to both abilities to resist urges to self-injure and intentions to self-injure. Although seemingly contradictory, it may be that participants with a strong intention to self-injure dismiss trigger warnings to view triggering content and dissuade themselves from engaging in the behavior. In so doing, individuals may feel more capable of resisting self-injury. Indeed, this speculation is congruent with findings from other work in which seeing or reading graphic content in web-based forums appears to assuage urges to injure [9,27]. The directionality of this relationship should be explored in future work.

Variance in viewing triggering content was negatively associated with the ability to resist self-injury. The more varied an individual’s viewing behavior was from day-to-day, the less they reported being able to resist self-injury. In contrast, the rate of change between proximal (the week before) and more distal (remaining time between surveys) viewing shows a negative relationship with the risk of self-injury behavior and intentions to injure. As the change score increased in magnitude (ie, more viewing in a week before a survey relative to a distal period), the likelihood of self-injury behavior and intention to self-injure decreased. Together, these findings reflect a nuanced relationship in which day-to-day variability in viewing triggering content is linked to poorer ability to resist urges—yet an increase in proximal to more distal viewing activity is related to less likelihood of self-injury and self-injury intentions. Variance reflects instability, so it may be that variance in viewing triggering content is characteristic of maladaptive coping. The change score represents a quantitative shift in weekly activity from more distal activity. Thus, from a prediction perspective, high rates of change should signal potential risk. Future work should probe this complex relationship more deeply.

### Key Findings on Language

In response to the second set of research questions related to language, we found (1) no relationship between affiliative language and self-injury outcomes, (2) that mentions of family were negatively related to intentions to injure, (3) that variance of positive emotions and anger were related to the ability to resist self-injury, and (4) that ruminative variance was related to self-injury thoughts.

In addition to these larger themes, self-referent language in posts was negatively associated with self-reported ability to resist self-injury. This finding is consistent with work showing that self-referent language is associated with poor mental health status [18,71,72]. In contrast, the use of efficacy language (eg, will, soon, always) was positively associated with the ability to resist self-injury. These findings echo previous work on the importance of self-efficacy in behavior change and recovery [73]. How confident individuals feel about their capacity to change can vary significantly as one’s relationship to self-injury changes [74,75]. This finding is promising because it provides further evidence for the role of efficacy in the ability to resist self-injury urges and provides some validity for the method of connecting language traces to self-report surveys.

Another key finding is that as familial language increased, intentions to injure decreased. This aligns with the literature citing family as a protective factor and family disharmony as a key risk factor for self-injury [76-78]. Understanding the link between familial language in web-based communication about self-injury and self-injury outcomes may be an important area for future research.

Patterns in emotional expressions were also observed for self-injury thoughts and the ability to resist. Specifically, participants were more likely to have thoughts of self-injury when posts in the week before a survey varied in the use of ruminative language. This is in line with other studies that found rumination instability predicted daily reports of NSSI [54]. In contrast, higher levels of variance in anger expressions were associated with a greater ability to resist self-injury. This finding is interesting in the absence of a main effect of anger. Many individuals who self-injure have trouble dealing with negative emotions, and anger is often cited as an affective state leading to self-injury [20]. It is possible that fluctuations in anger (or an ebb and flow of this affective experience) signal healthier adaptive processing or simply an ability to express anger and a variety of other emotions.

Finally, we found that as the rate of change of positive emotion increased, so did the ability to resist self-injury. Expressions of positive emotion have been associated with improved well-being in previous work [66], and we find that more positive emotion in proximal—relative to distal periods—is associated with a greater ability to resist.

### Implications

The findings highlight implications for researchers working in digital mental health, clinicians working with young people who self-injure, and platform designers. In terms of research, much of the previous work investigating web-based communication about self-injury has focused on “static entities such as websites and forums rather than the fragmented, heterogeneous and dynamic current landscape of social media” [79]. This study uses a mixed methodological approach to capture the dynamic nature of activity on a mobile peer-support app in relation to how individuals manage urges and self-injury behavior in their daily lives. Although we focus on methodological limitations in the next section, we feel that the dynamicity captured in this study is a key strength that can be leveraged in future investigations. Lagged regressions and studies using platform-initiated push notifications may be particularly useful extensions of this work to further disentangle bidirectional temporal relationships and identify opportunities for timely intervention. The factors identified through this work, particularly those related to web-based behaviors and shifts in self-injury behavior over time, if replicated, could inform future modeling to identify users who may be in need of additional resources.

Our findings also emphasize the importance of considering web-based activity, as it relates to self-injury recovery in the context of treatment [30,34,80]; 42% of our participants reported having been in therapy at some point throughout the study, yet clinical assessments do not routinely probe for web-based engagement [30]. When clinicians are not aware of their clients’ web activity, they risk overlooking critical aspects of clients’ social environment and sources that likely shape their motivation, or ambivalence, toward self-injury behavior change.

Specifically, our findings suggest that clinicians should check in with clients regarding posting and viewing self-injury content. Posting content labeled as triggering was related to self-injury behavior and thoughts in our study, and viewing such content was related to both greater ability to resist self-injury and greater intentions to injure. Of note, we found that exposure to such content is not necessarily related to greater self-injury frequency. Although these findings are preliminary, they are in line with other previous work, suggesting that conventionally *risky* activities may not always be harmful [79]. This relationship between exposure to content and effects is nuanced and likely depends on individual factors that are not well captured in our data but may surface in the context of a therapeutic relationship. Clinicians should strive for a nuanced understanding of the client’s motivation to post and view content and react to client disclosures of web-based engagement in a curious but nonjudgmental way to encourage further dialog. As young people turn to digital tools to manage self-injury urges and related factors (eg, mood and interpersonal relationships) between treatment sessions, it is imperative that clinicians have a basic understanding and awareness of what resources exist and how clients use them. This awareness will enable clinicians to ask specific and relevant questions and regularly assess the impact web-based activities have on the client’s progress toward recovery. Several useful guides have been designed to assess web activity in sessions with individuals who self-injure and could be adapted to reflect specific features of new platforms such as TalkLife [30,34].

Our findings also have relevance for platform designers. As confidence in being able to identify individuals engaging in, or at risk of, self-injury increases, we can begin to think about how to deliver timely and accessible interventions. Given that most of our participants were not currently in therapy, it may be beneficial to consider integrating elements of evidence-based treatment into the platform experience (eg, psychoeducation). These elements could be optional and be offered based on the individual’s use patterns. Finally, although the tendency has been to remove graphic, or potentially triggering, content from platforms, there have been discussions about the dangers of overmoderation [79,81,82] and the benefits of self-expression [83]. Our findings suggest a need to consider safe ways of moderating content while also allowing free expression. Balancing user agency within systems that encourage positive behaviors is a challenge, but a worthy endeavor, as there is ample evidence that distressed individuals use online spaces to solicit support and connect with the community.

### Limitations

This study has several limitations that should be acknowledged. First, the methodological design imposes limitations on causality. Survey questions were framed to ask about any self-injury in the week before but did not ask about specific days, or times of day, when these events (eg, thoughts, behaviors) occurred. Therefore, it was not possible to establish a detailed timeline for when the self-injury events occurred relative to the activities on TalkLife. Future work might consider daily surveys, either through diary or EMA, for a more nuanced understanding of these temporal relationships. We also chose to aggregate our data at the week level in this study, but it would be worthwhile for future work to explore associations between self-injury outcomes and web-based activity across other periods (eg, daily, monthly).

Second, while LIWC is widely used for language analysis, it does not account for context. Several key findings on language are tentative and should be more thoroughly explored in future work. One way to do this would be to triangulate with other language analysis techniques such as the tf-idf or word co-occurrence measures derived from n-grams.

Third, we operationalized web-based activity as *active* use. Owing to high correlations between active and passive measures in this data set, we restricted the analysis to active log data to avoid collinearity issues. However, research has shown that active and passive social media use can have differential effects on affective well-being [84,85]. Thus, future work should explore the influence of a more comprehensive spectrum of engagement on self-injury outcomes.

There are limitations to the generalizability of these findings. TalkLife is a platform specifically intended for the exchange of support related to mental health challenges, and the extent to which our findings generalize to other social platforms is unknown. In addition, there is potential for selection bias in this study. Participants were inclined to use support apps, willing to take weekly surveys on their self-injury, and engaged in active use of the app. This suggests that participants may have been similar in ways related to their use and their readiness to change self-injury behaviors. Future work may wish to consider recruiting and incentivizing a more diverse population for a broader picture of TalkLife activity.

Our findings should also be contextualized in light of the limitations of our analytical approach. Although variable selection procedures are common in psychological and social scientific research, alternative approaches, such as stochastic search variable selection or lasso regression, may enhance the reliability of models when selecting variables from a large number of predictors [86]. We note that these approaches would be useful in future work.

Finally, this exploratory work was meant to identify factors associated with self-injury outcomes that could be targeted in future confirmatory research with sufficiently powered samples. The methodological approach of combining naturally occurring web-based data with self-report survey data provides new insights into the relationship between the use of a web-based peer-support platform and various self-injury outcomes.

### Conclusions

This study investigated the relationship between web-based support activities and self-injury outcomes to identify patterns that may be beneficial or harmful. To do so, we employed a novel mixed methods approach that utilized naturally occurring language and behavioral data from a mobile peer-support app and survey data collected over 4 months. Our findings point to a nuanced set of relationships. Specifically, participants who actively engaged in TalkLife were at lower odds of reporting self-injury thoughts and intentions. However, activity level was not predictive of self-injury behavior or the ability to resist self-injury urges. Posting triggering content was associated with greater odds of participants reporting self-injury thoughts and behaviors, whereas viewing triggering content was linked to both greater abilities to resist urges and greater intentions to injure. This work provides empirical support for the relationship between participation in a web-based support platform and 5 self-injury outcomes and articulates patterns that merit consideration in future work. We hope that insights from this study will inform future research on digital mental health and platform design.

## Abbreviations

EMA: ecological momentary assessment
LIWC: Linguistic Inquiry And Word Count
NSSI: nonsuicidal self-injury
OR: odds ratio
RQ: research question
VIF: variance inflation factor
